# Whole-genome analysis for effective clinical diagnosis and gene discovery in early infantile epileptic encephalopathy

**DOI:** 10.1038/s41525-018-0061-8

**Published:** 2018-08-13

**Authors:** Betsy E. P. Ostrander, Russell J. Butterfield, Brent S. Pedersen, Andrew J. Farrell, Ryan M. Layer, Alistair Ward, Chase Miller, Tonya DiSera, Francis M. Filloux, Meghan S. Candee, Tara Newcomb, Joshua L. Bonkowsky, Gabor T. Marth, Aaron R. Quinlan

**Affiliations:** 1 Division of Pediatric Neurology, Department of Pediatrics, University of Utah School of Medicine, Salt Lake City, UT USA; 2 Department of Human Genetics, University of Utah School of Medicine, Salt Lake City, UT USA; 3 Department of Biomedical Informatics, University of Utah School of Medicine, Salt Lake City, UT USA

## Abstract

Early infantile epileptic encephalopathy (EIEE) is a devastating epilepsy syndrome with onset in the first months of life. Although mutations in more than 50 different genes are known to cause EIEE, current diagnostic yields with gene panel tests or whole-exome sequencing are below 60%. We applied whole-genome analysis (WGA) consisting of whole-genome sequencing and comprehensive variant discovery approaches to a cohort of 14 EIEE subjects for whom prior genetic tests had not yielded a diagnosis. We identified both de novo point and INDEL mutations and de novo structural rearrangements in known EIEE genes, as well as mutations in genes not previously associated with EIEE. The detection of a pathogenic or likely pathogenic mutation in all 14 subjects demonstrates the utility of WGA to reduce the time and costs of clinical diagnosis of EIEE. While exome sequencing may have detected 12 of the 14 causal mutations, 3 of the 12 patients received non-diagnostic exome panel tests prior to genome sequencing. Thus, given the continued decline of sequencing costs, our results support the use of WGA with comprehensive variant discovery as an efficient strategy for the clinical diagnosis of EIEE and other genetic conditions.

## Pediatric neurology: whole-genome analysis finds mutations causing severe newborn seizures

Whole-genome sequencing combined with specialized bioinformatics can diagnose disease mutations in newborns with devastating seizures. Josh Bonkowsky, Gabor Marth, Aaron Quinlan, and colleagues from the University of Utah in Salt Lake City, USA, thoroughly detailed the genomic variation of 14 babies with early infantile epileptic encephalopathy (EIEE). EIEE is a severe pediatric neurodevelopmental disorder causing seizures and early death. In the 14 infants, who had previous testing that did not identify the cause, the research team found the genetic cause for all of the infants. Prior to this work, approaches for EIEE diagnosed only up to 60% of infants. With the cost of DNA sequencing continuing to fall, the authors suggest that whole-genome testing could be a cost-effective approach to diagnosing EIEE and other genetic conditions.

## Introduction

Early infantile epileptic encephalopathy (EIEE) is a rare epilepsy syndrome that causes intractable seizures with multiple seizure types and presents in the first months of life. While the prevalence of EIEE is unclear, and only affects a subset of all infants with seizures (about 1.2 of 1000 live births),^1^ infants with EIEE have serious medical complications. EIEE patients typically exhibit developmental delay, profound intellectual impairment, and progress to severe psychomotor impairment and early death.^2,3^ Structural brain malformations, birth injury, and inborn errors of metabolism can cause EIEE, but once these causes are accounted for most remaining cases of EIEE are presumed to have a genetic basis. Structural variation (SV) such as large deletions or duplications are identifiable by karyotype or chromosomal microarray studies, and account for a relatively small proportion of cases, with estimates between 6 and 18% in recent studies.^4,5^ Mutations in more than 50 different genes^6^ have been described in EIEE. While gene panel and whole-exome sequencing approaches have been used in EIEE, diagnostic yields are no higher than 60%.^7–9^ Since diagnostic testing by traditional means can be expensive and continue for years,^10–12^ improving the speed and reducing the cost associated with genetic tests would have substantial clinical impact.

## Results

### Subject cohort and sequencing

From 2015 to 2016, we recruited subjects with EIEE for whom no underlying diagnosis was identified despite extensive prior testing. We excluded subjects with established genetic, metabolic, structural, or birth trauma-related causes. The final cohort included 14 subjects for whom DNA was also available from both parents (see Table 1 and Supp. Table 1 for extensive phenotypic and prior testing information for these subjects). We anticipated that, for the majority of subjects, the causative variant would be a de novo mutation,^13–16^ which are notoriously difficult to detect accurately from short-read sequencing data.^17^ Therefore, we performed deep whole-genome Illumina sequencing on all 14 families (i.e., 42 individuals). Two sequencing lanes from two distinct DNA libraries were used to maximize discovery in each family, producing an average of 65× (range 51× to 93×) median coverage per individual (see Supp. Table 2 and Supp. Fig. 1). Increased sequence coverage provides greater power to detect de novo mutations in subjects, and it also reduces false positive de novo mutation predictions in cases where the transmitted allele is not sequenced in one of the parents.^17,18^

**Table 1:** Summary of clinical phenotypes and prior genetic testing for each EIEE subject

Subject	Gender	Age at onset	Clinical details	Prior genetic testing (all results normal)
1	M	<1 mos.	Seizure types: Generalized tonic, myoclonic Clinical features: GDD, hypotonia, dysphagia, hydronephrosis, EEG features: Slow and disorganized background, multifocal and generalized SW discharges MRI: Abnormal T1 signal in basal ganglia, mesial temporal lobes	Chromosomal microarray, STXBP1*, MECP2*^, ARX*^, CDKL5*
2	F	2 mos.	Seizure types: GTC, generalized tonic, myoclonic, flexor spasms, atonic Clinical features: GDD, dysphagia, cortical visual impairment, microcephaly EEG features: Slow and disorganized background, generalized SW discharges MRI: Normal	Chromosomal microarray, SLC2A1*
3	F	<1 mos.	Seizure types: Generalized tonic, flexor spasms Clinical features: HIE, GDD, chronic respiratory failure, spasticity, dysphagia, cardiac arrest EEG features: Slow background, frontal SW discharges MRI: Abnormal diffusion restriction bifrontal and left temporal	Chromosomal microarray
4	M	<1 mos.	Seizure types: Generalized tonic Clinical features: Hypotonia, GDD, cerebral palsy, dysphagia EEG features: Slow background, multifocal and generalized SW discharges MRI: Delayed myelination	*PLP1^*
5	F	2 mos.	Seizure types: Focal onset with secondary GTC Clinical features: GDD, postnatal microcephaly, spastic quadriparetic CP, chorea, dystonia EEG features: Focal slowing MRI: Normal	Chromosomal microarray, STXBP1*^, *CLN2*^*+*^*, CLN3*^*+*^, ARX*^, Early Infantile Epilepsy Panel, Febrile Seizures Panel
6	F	<1 mos.	Seizure types: Migrating partial seizures, myoclonic, flexor spasm Clinical features: GDD, dysphagia, quadriparetic spastic cerebral palsy EEG features: Generalized and multifocal SW discharges MRI: Normal	Chromosomal microarray, DNA methylation, MECP2*, CDKL5*
7	M	1 mos.	Seizure types: Generalized clonic seizures, GTC, flexor spasms, tonic spasms Clinical features: GDD EEG features: Slow background, multifocal and generalized SW MRI: Normal	None
8	F	<1 mos.	Seizure types: GTC, focal tonic Clinical features: GDD, hypotonia, cerebral palsy, postnatal microcephaly EEG features: Discontinuous, multifocal SW MRI: Normal	MECP2*, ARX*, DNA methylation, Early Infantile Epilepsy Panel, Comprehensive Epilepsy Panel
9	F	6 mos.	Seizure types: Myoclonic, atonic, myoclonic, partial seizures, GTC, atypical absence Clinical features: Postnatal microcephaly, hypotonia, GDD, polymyoclonus EEG features: 1-2hz delta, generalized SW MRI: Delayed myelination	Chromosomal microarray, DNA methylation, MECP2*^, Rett-like Disorders Panel, Comprehensive Epilepsy Panel
10	M	4 mos.	Seizure types: Flexor spasms, tonic spasms, GTC Clinical features: GDD, spastic cerebral palsy, postnatal microcephaly EEG features: Hypsarrhythmia, multifocal SW MRI: Delayed myelination	Chromosomal microarray, ARX*
11	F	2 mos.	Seizure types: Migrating focal tonic clonic, generalized tonic, myoclonic, atonic Clinical features: GDD, macrosomia, tremor, hypotonia EEG features: Focal SW discharges MRI: Abnormal periventricular white matter T2 signal	Chromosomal microarray
12	M	6 mos.	Seizure types: Generalized tonic spasms, atonic Clinical features: GDD, hypotonia, constipation EEG features: Slow and disorganized background, multifocal and generalized SW MRI: Normal	None
13	F	4 mos.	Seizure types: Hemiclonic, prolonged febrile, GTC, focal tonic Clinical features: Language delay EEG features: Focal slowing, generalized SW MRI: Normal	SCNA1*
14	F	<1 mos.	Seizure types: GTC, absence, complex partial Clinical features: GDD, hypotonia, dysphagia, spasticity EEG features: Slow and disorganized background, suppression burst MRI: Normal	Chromosomal microarray, DNA methylation, SCN1A*

*GTC* generalized tonic clonic, *GDD* global developmental delay, *HIE* hypoxic ischemic encephalopathy, *SW* spike wave

*Sequencing of individual gene

^Deletion/duplication analysis

^+^Enzyme testing

### Variant identification

After sequence alignment with BWA-MEM,^19^ we carried out comprehensive detection of genetic variation in each EIEE family trio, using a combination of existing alignment-based tools and our reference-free approach (Methods). We scanned each family for single-nucleotide variants (SNVs) and insertion-deletions (INDELs) using the GATK^20^ best practices pipeline. We also used LUMPY^21^ to detect structural variants (SV) and copy number variants (CNV), in conjunction with SVTyper^22^ to generate SV genotypes for each family member. Because of the strong prior expectation that the causative variant would be a de novo mutation in the affected child, we also applied RUFUS,^23^ our k-mer-based, alignment-free analysis algorithm designed specifically to reduce false positive de novo mutations predictions (see Methods) and reveal mutations that can be missed by alignment-based approaches.

### Variant prioritization

With candidate de novo mutations detected in the 14 probands, we followed a tiered variant prioritization strategy to identify causative mutations (see Table 2). We first targeted missense, frameshift, or nonsense coding mutations within known genes associated with EIEE using both GEMINI^24^ and the web-based variant visualization and interrogation tool gene.iobio (http://gene.iobio.io). GEMINI was used to identify de novo mutations in genes that ClinVar^25^ associated with the terms “epileptic” and “infant”. To prioritize variants with gene.iobio, we first created an inclusive list of 223 EIEE candidate genes (Supp. Table 4) by merging genes across EIEE-specific gene panel tests and ClinVar,^25^ followed by a Phenolyzer^26^ search with the relevant phenotype search terms (see Methods). Candidate variants were classified as “pathogenic” or “likely pathogenic” according to ACMG criteria.^27^

**Table 2:** Mutations and affected genes identified for each subject

Subject	Gene	Variant	ACMG variant classification^a^	Genomic location	Novel gene/known EIEE gene/previously reported
1	*KCNQ2*	c.841G>A, p.G281R	Pathogenic (PS1, PS2, PM2, PP3, PP5)	chr20:62071037	Known gene, previously reported mutation^43,55^
2	*Multiple*	Balanced inverted translocation between Chr2 and ChrX		chrX:151118513 chr2:59405748	Novel structural mutation similar to previously structural rearrangement^42^
3	*SCN8A*	c.1219T>G, p.L407V	Pathogenic (PS2, PM2, PM5, PP2, PP3)	chr12:52099285	Known gene, previously reported mutation at this site^56^
4	*PIGA*	c.502A>C, p.N168H	Likely pathogenic (PS2, PM2, PP3)	chrX:15349551	Known gene, novel mutation
5	*SCN8A*	c.800T>C, p.L267S	Likely pathogenic (PS2, PM2, PP2, PP3, PP5)	chr12:52093447	Known gene, novel mutation (same patient and mutation reported concurrently in Malcolmson et al.^57^)
6	*EEF1A2*	c.1267C>T, p.R423C	Pathogenic (PS1, PS2, PM2, PP3)	chr20:62119776	Known gene, previously reported mutation^58^
7	*CDKL5*	c.146-14735_2276+3273dup	Pathogenic (PVS1, PS2, PM2, PP3, PP5)	chrX:18567862 -18630963	Known gene with previously reported loss of function mutations^28–30^
8	*SCN2A*	c.647T>G, p.L216W	Likely pathogenic (PS2, PM2, PP2, PP3)	chr2:166165903	Known gene, novel mutation
9	*DEAF1*	c.634G>A, p.G212S	Pathogenic (PS1, PS2, PS3, PM2, PP3, PP2)	chr11:687941	Known gene, previously reported mutation^33^
10	*CAMK2G*	c.719C>T, p.T240M	Likely pathogenic (PS2, PM2, PP2, PP3)	chr10:75607083	Novel gene
11	*STXBP1*	c.1151dup, p.D385Gfs*19	Pathogenic (PVS1, PS2, PM2, PP3)	chr9:130438123	Known gene, previously reported loss of function mutations^33,43^
12	*SCN8A*	c.2642T>C, p.V881A	Likely pathogenic (PS2, PM2, PP2, PP3, PP5)	chr12:52159552	Known gene, novel mutation
13	*SCN1A*	c.4736T>C, p.L1590P	Likely pathogenic (PS2, PM2, PP2, PP3)	chr2:166850739	Known gene, novel mutation
14	*KCNQ2*	c.833T>C, p.I278T	Likely pathogenic (PS2, PM1, PM2, PP3)	chr20:62071045	Known gene, novel mutation

^a^PVS1—Null variant (nonsense, frameshift, canonical ±1 or 2 splice sites, initiation codon, single or multi-exon deletion) in a gene where LOF is a known mechanism of disease

PS1—Same amino acid change as a previously established pathogenic variant regardless of nucleotide change

PS2—De novo (both maternity and paternity confirmed) in a patient with the disease and no family history

PS3—Well-established in vitro or in vivo functional studies supportive of a damaging effect on the gene or gene product

PS4—Prevalence of the variant in affected individuals is significantly increased compared with the prevalence in controls

PM1—Located in a mutational hot spot and/or critical and well-established functional domain (e.g., active site of an enzyme) without benign variation

PM2—Absent from controls (or at extremely low frequency if recessive) in Exome Sequencing Project, 1000 Genomes Project, or Exome Aggregation Consortium

PM5—Novel missense change at an amino acid residue where a different missense change determined to be pathogenic has been seen before

PP2—Missense variant in a gene that has a low rate of benign missense variation and in which missense variants are a common mechanism of disease

PP3—Multiple lines of computational evidence support a deleterious effect on the gene or gene product (conservation, evolutionary, splicing impact, etc.)

PP5—Reputable source recently reports variant as pathogenic, but the evidence is not available to the laboratory to perform an independent evaluation

In 9 of the 14 subjects, GEMINI identified a single, de novo variant with high confidence in pathogenicity. Of these, seven subjects carried de novo missense variants in ion-channel genes (*SCN1A*, *SCN2A*, *SCN8A*, *KCNQ2*) with known association to EIEE (Table 2, Supp. Table 5). One subject had a de novo missense variant in the eukaryotic translation elongation factor 1 alpha 2 (*EEF1A2*) gene, and another subject harbored a one base pair frameshift insertion in the syntaxin-binding protein 1 (*STXBP1*) gene. In addition, gene.iobio identified a likely pathogenic mutation in a tenth subject within the phosphatidylinositol glycan anchor biosynthesis class A (*PIGA*) gene. Notably, these procedures allowed us to rapidly (in less than 5 min) screen a comprehensive candidate gene list and identify diagnostic variants in EIEE-associated genes for 10 of the 14 subjects (subjects #1, 3–6, 8, 11–14).

**Fig. 1:**
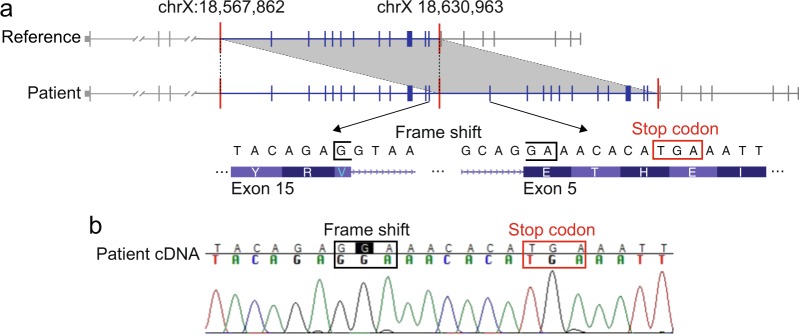
**a** A 63 kb de novo tandem duplication in *CDKL5* duplicates exons 5 through 15 (for Ensembl canonical transcript ENST00000379989) in subject 7. **b** Targeted cDNA sequencing confirms the predicted frameshift and stop gain mutation caused by the de novo tandem duplication

For the remaining four subjects, we searched for de novo SVs predicted to disrupt genes that have been previously implicated in EIEE. In subject #7, we detected a 63 kb de novo duplication within *CDKL5*. This copy number mutation created a tandem duplication of exons 5 through 15 (Fig. 1a) that we predicted to cause a frameshift when splicing of the mutant transcript joins exon 15 with the duplicated exon 5. In turn, the frameshift is predicted to create a stop codon five amino acids downstream from the end of the first copy of exon 15. The tandem duplication, frameshift, and stop gain were confirmed by sequencing cDNA derived from a fresh blood sample from subject #7 (Fig. 1b). This mutation is predicted to have an X-linked recessive effect in our male patient in a gene previously associated^28–30^ with EIEE.

For subjects #2, #9, and #10, we then searched for de novo missense or putative loss of function (i.e., nonsense, frameshift, and splice donor/acceptor) mutations in protein coding regions of genes not previously associated with EIEE. This search led to 18, 22, and 12 GATK-called variants, and 0, 1, and 2 RUFUS-called variants, respectively. Manually excluding low-quality variant calls and reviewing the potential for association with the phenotype, we excluded all but a single de novo variant (i.e., the one called by RUFUS) in subject #9, in the DNA binding, SAND domain of the *DEAF1* gene. Missense variants in the SAND domain of DEAF1 have been previously reported in association with dominant intellectual disability phenotypes, and a severe recessive epilepsy phenotype.^31,32^ The same allele identified in subject #9 (p.G212S) was recently reported in a 15-year-old male with developmental regression and seizures.^33^ Functional studies suggest that this allele eliminates both DEAF1 transcriptional repression activity and DEAF1–DNA interactions.

Subject #10 harbored a de novo missense variant in *CAMK2G*, the gamma subunit of the calcium/calmodulin-dependent protein kinase II (CAMKII) complex. CAMKII is a multi-subunit complex that plays an essential role in synaptic function including learning and memory.^34^ The alpha and beta isoforms (*CAMK2A* and *CAMK2B*) are involved in calcium signaling in glutamatergic synapses.^35^ Furthermore, the CAMKII complex has been implicated in temporal lobe epilepsy,^36^ and de novo mutations in *CAMK2A* and *CAMK2B* were reported to cause intellectual disability.^37^ The variant identified in our subject substitutes a threonine with methionine in a highly conserved region of the catalytic subunit of *CAMK2G*. This variant is extremely rare: it is observed as a heterozygote in only one Finnish individual of >138,000 individuals sequenced in the gnomAD database,^38^ and incomplete penetrance could explain the lack of a known seizure phenotype for the gnomAD individual. While not directly associated with epilepsy or other clinical phenotypes, *CAMK2G* has been predicted to be a drug target for refractory epilepsies.^39^ A separate de novo variant in *CAMK2G* (c.1075G>A, p.V359M) was observed in a developmental disorder proband as part of the DDD study,^40^ but pathogenicity details of the phenotype were not available.

**Fig. 2:**
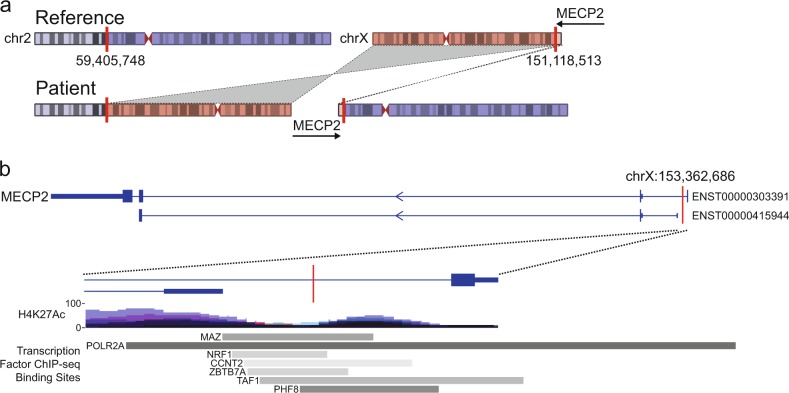
An inverted, reciprocal translocation between chromosomes X and 2. **a** The inverted translocation in subject 2 results in DNA exchange between the X chromosome and chromosome 2. The chromosome 2 break occurred in the p arm at position 59,405,748, leaving minor (24%) and major (76%) portions, and the chromosome X break occurred at the extreme q arm at position 151,118,513 leaving a minor (3%) and major (97%) portions. As a result, GABRE, GABRA3, and MECP2 are translocated from the X chromosome to chromosome 2. **b** A de novo mutation in subject 2 is also observed that is intronic to multiple isoforms (e.g., ENST00000303391) of MECP2 and upstream of other isoforms (e.g., ENST00000415944) of MECP2. The mutation lies within the observed binding site of multiple transcription factors, including Pol II

Lastly, for subject #2 we identified a de novo, inverted, balanced translocation between chromosome 2p16.1 and chromosome Xq28 (Fig. 2). This rearrangement moves a short, but gene-dense segment of chromosome X to chromosome 2. The translocated segment of chromosome X includes 92 genes with a breakpoint between *MAGEA4* and *GABRE*. In this segment, three genes coding for subunits of the GABA receptor genes (*GABRE*, *GABRA3*, and *GABRQ*) and *MECP2* have potential neurological phenotypes. Other GABA receptor genes including *GABRA1*, *GABRB1*, and *GABRB3* have been associated with severe epilepsy phenotypes.^41^ While we did not find a sequence variant associated with epilepsy in this subject, the translocation likely disrupts patterns of X-inactivation and alters transcription patterns.^42^ Furthermore, *MECP2* is associated with Rett syndrome and is approximately 2 Mb from the translocation breakpoint. There is some phenotypic similarity between subject #2 and patients with Rett syndrome, including microcephaly, seizures, and developmental regression. Furthermore, a Rett syndrome phenotype was described in a previous patient^42^ with a pericentric inversion in the vicinity of *MECP2*. We also identified a de novo variant in subject #2 that impacts an intronic or upstream (depending on the isoform) *POL2* binding site within *MECP2*, though it is unclear if there is a change in transcript level as a result of this variant. Given the known association between MECP2 and infantile seizure disorders, as well as the Rett-like phenotype of this subject, we hypothesize that the disruption of *MECP2* transcription is the most plausible mechanism.

**Fig. 3:**
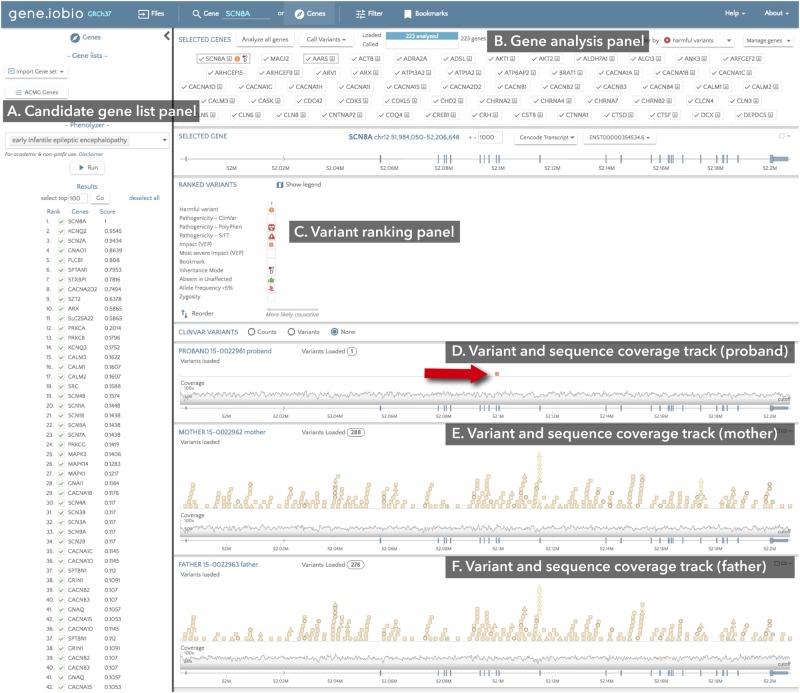
Gene.iobio screenshot of the diagnostic de novo variant in subject #3, detected in the SCN8A gene. **a** Candidate gene panel, in this example displaying the phenotype-driven EIEE candidate gene list generated by the integrated Phenolyzer tool. **b** Gene analysis panel showing the status and results of the analysis on all candidate genes. Analyzed genes are sorted by the most likely causative variant, resulting in the SCN8A at the top of the list. **c** Variant ranking panel, displaying the single, non-synonymous de novo mutation in gene SCN8A (indicated by the red arrow). **d** Variant and sequence coverage track for the proband. Based on the filters selected in the filtering panel (not shown), only de novo mutations in the gene are shown, in this example a single variant marked by the red arrow. **e** Variant and sequence coverage track in the proband’s mother, showing all variants, inherited and de novo, in this sample. **f** Variant and sequence coverage track in the proband’s father, showing all variants, inherited and de novo, in this sample

This study represents the first diagnostic application of our RUFUS de novo mutation detection method^23^ (manuscript in preparation). In contrast to the read alignment-based variant detection methods that are most commonly used today, the alignment-free, k-mer-based RUFUS algorithm directly compares k-mers in the sequencing reads between a child and his/her parents to identify child-specific k-mers that suggest de novo mutations. This strategy avoids the vast majority of the false positive mutation calls that arise from read alignment artifacts in alignment-based methods. Therefore, the main advantage of RUFUS over alignment-based detection approaches is the much higher specificity for calling mutations. For example, RUFUS detected on average 1.7 coding de novo mutations per subject, as compared to the average of 61.8 de novo mutation detected by GATK (Supp. Table 3). In fact, in 6 of the 14 subject genomes, RUFUS only called a single coding variant, and in 7 of the 14 subjects only a single-amino acid-changing variant (see Fig. 3 for an example). Furthermore, RUFUS detects all forms of de novo mutation in a single step, including SNVs, short INDELs, and SVs, thereby eliminating the need to run multiple detection programs on the data. RUFUS detected all diagnostic and putative disease causing mutations uncovered in this study, while reporting only a handful of additional mutations affecting coding sequences.

## Discussion

Currently, clinical diagnosis of EIEE is not standardized, and can include radiological imaging, metabolic testing, and genetic testing ranging from single gene tests, to panel testing or whole-exome sequencing.^10,12^ However, many subjects remain undiagnosed, leading to prolonged and expensive diagnostic odysseys. The increasing availability of high-throughput DNA sequencing has led to an increased number of EIEE patients with genetic diagnoses. In a recent study of a similar cohort of infants with epileptic encephalopathies, a definitive genetic diagnosis was reached in ~60% of infants using a combination of epilepsy gene panels and whole-exome sequencing.^43^ Here, we identified a genetic diagnosis in all 14 subjects using comprehensive WGA that includes identification of both sequence variants and SVs. Our results suggest that early implementation of methods interrogating spontaneous SVs, SNVs, and INDELs are necessary for comprehensive EIEE diagnosis.

Furthermore, despite the current cost of clinical WGS (typically currently ranging from $5000 to $15,000 per trio),^44,45^ it is more cost and time effective than current diagnostic approaches. In our cohort, the oldest child was more than 16 years of age when a diagnosis was finally determined. Each subject received a minimum of 24 diagnostic tests (Supp. Table 6), resulting in average charges of $30,866 (range $16,592–$50,348) prior to whole-genome sequencing. Overall, charges in pursuit of diagnosis for the entire cohort was $432,121. Clinical whole-genome costs are still higher than gene panels or whole-exome sequencing. However, whole-genome sequencing as an initial diagnostic strategy offers a potential time and cost-savings approach, and a more comprehensive single-step evaluation of non-coding and CNVs, compared to the standard approach of multiple, sequential tests. In addition, while data processing and analysis requirements are substantial, the turn-around time and cost of all high-throughput sequencing approaches continues to drop, enabling whole-genome sequencing to be employed in a clinical setting with a turn-around of 14 days or less.^46^

In an effort to find a genetic diagnosis for each subject, we sought greater than typical (~65×) sequencing depth per sample, motivated by earlier observations^17^ that the accuracy of detecting de novo variants with mapping-based detection approaches improves with greater coverage. Using deep whole-genome sequencing, we identified pathogenic or likely pathogenic variants, as defined by the ACMG guidelines, in 12 of 14 cases; and likely diagnostic variants in the remaining 2 of 14 subjects in our cohort. These variants were confirmed by Sanger sequencing. The high specificity of variant detection is evidenced by the fact that, for 10 of the 14 subjects, both our mapping-based and reference-free methods only detected a single coding mutation in a known EIEE-associated gene, and, in each case, this was the diagnostic variant.

Since 12 of the 14 subjects harbored SNV or INDEL mutations in EIEE-associated genes, it can be argued that exome sequencing is a rational diagnostic alternative to whole-genome sequencing. We note, however, that patients #5, #8, and #9 had been previously tested with commercial, exome-based gene panels which failed to report the mutations we observed with whole-genome sequencing. Furthermore, we emphasize that whole-genome sequencing was critical to the discovery of diagnostic variants in two of our cases. Neither the precise structure and consequence of the *CDKL5* tandem duplication in subject #2, nor the reciprocal translocation in subject #7, could be characterized with gene panels or exome sequencing. Finally, the detection of a potentially regulatory de novo mutation in *MECP2* was only possible using whole-genome sequencing for subject #2.

Given the continued improvements in the cost and speed of whole-genome sequencing, we argue that, in the next few years, whole-genome sequencing approaches are likely to become the standard approach to arriving at a definitive EIEE diagnosis. In general, whole-genome sequencing offers particular advantages for clinical diagnosis of monogenic diseases with well-defined phenotypes, where the majority of causative genes are largely known. Polygenic disorders or diseases with substantial environmental risk factors are less amenable to diagnosis with WGS owing to the complexity of confidently identifying the set of genetic changes driving the phenotype. Our high diagnosis rate was facilitated by the availability of parental DNA for all subjects; the existence of a fairly well-defined set of candidate genes involved in epilepsy; having sufficient sequencing depth to confidently detect de novo mutations; the use of complementary tools for the discovery of all forms of genetic variation; and the fact that EIEE patients are enriched for pathogenic de novo mutations. We anticipate that whole-genome sequencing will ultimately provide improvements in the diagnosis of a broad range of other genetic disorders, including leukodystrophies, skeletal dysplasias, and congenital cardiac diseases.

## Methods

### Cohort assembly

This study was approved by the Institutional Review Boards of University of Utah. We assembled a retrospective cohort of subjects followed by the Pediatric Neurology Division at the University of Utah through their outpatient clinics at Primary Children’s Hospital (PCH) between 2015 and 2016, who were born between 2004 and 2016. We reviewed history and EEG findings to confirm the diagnosis of EIEE. We also reviewed MRI and laboratory data to confirm that subjects did not have an inborn error of metabolism, an established genetic diagnosis, or a structural brain abnormality.

### Cohort consent

The study was approved by the Institutional Review Boards of the University of Utah and PCH. Written informed consent was obtained from the parents of the patients. After obtaining informed consent, proband and parents were enrolled in the study.

### Sequencing

DNA was extracted from blood or saliva. Genomes of the 42 study individuals were prepared using TruSeq DNA PCR-free libraries (Illumina) and run on the Illumina HiSeq X Ten System at a minimum of 60× median whole-genome coverage.

### Sequence processing

Sequence reads were aligned to the GRCh37 reference genome (including decoy sequences from the GATK resource bundle) using the BWA-MEM^19^ read alignment program. BAM files were de-duplicated with samblaster.^22^ INDEL realignment and base quality recalibration was performed using the GATK package.^47^

### Quality control

After alignment we evaluated global coverage using indexcov^48^ and verified that there were no major anomalies, and that the relative coverage levels on the sex chromosomes matched the expected sexes. After variant calling was completed, we ran peddy^49^ to verify that the relationships inferred from the genotypes matched those reported in the pedigree information and to evaluate depth of coverage in variant calls and ancestry composition. We also utilized mosdepth^50^ to extract per-base coverage and to estimate sequence coverage for each sample (Supp. Table 2).

### Variant identification using mapping-based variant calling methods

Single-nucleotide and INDEL variant calling and genotyping was performed with the GATK Haplotype Caller.^47^ The resulting variant calls included both inherited and de novo variants (in the probands). SV identification was performed using the LUMPY program.^21^ The SVTyper subprogram was used to genotype each sample at each called SV candidate site. The resulting variant calls included both inherited and de novo variants in the probands.

#### De novo variant prioritization

GEMINI^24^ version 0.19.0 was used to identify high confidence, single-nucleotide, and INDEL de novo mutations for which each member of a family trio had a sequencing depth of at least 15 and a genotype quality of at least 10. Candidate mutations were also required to be predicted to have an impact severity of “MED” or “HIGH” in GEMINI (i.e., “missense”, “frameshift”, “stop_gained”, “stop_lost”, etc; see http://gemini.readthedocs.io/en/latest/content/database_schema.html#details-of-the-impact-and-impact-severity-columns for details) and to have an allele frequency of no greater than 0.001 in any of the sub populations in the ExAC^38^ database. Predicted impacts on protein function were annotated with VEP^38,51^ before the creation of the GEMINI database. Finally, candidate de novo mutations were restricted to those that ClinVar^25^ associated with disease terms “infant” and “epileptic”. The following command was used to identify candidate de novo mutations.

gemini de_novo -d 15 --min-gq 10 --columns “chrom, start, end, ref, alt, is_lof, codon_change, aa_change, gene, impact, clinvar_gene_phenotype, max_aaf_all” --filter “impact_severity! = ‘LOW’ and max_aaf_all < 0.001 and call_rate >= 0.95 and aaf < 0.05 and clinvar_gene_phenotype like ‘%epileptic%’ and clinvar_gene_phenotype like ‘%infant%’” Projects/eiee/ostrander-eiee.sav.db

Candidate de novo structural mutations were identified by screening the LUMPY SV predictions in each family for variants where the proband had a variant genotype, and both parents had homozygous reference genotypes. We further required the proband to have at least 15 alignments (paired-end or split-read) supporting the de novo SV and each parent to have zero supporting reads. We then removed SVs where either end overlapped a simple repeat defined by the UCSC genome browser for build 37 of the human genome. After these steps, only two SVs remained: the translocation in family 42,610, and the duplication in family 44,133.

### De novo variant identification using our RUFUS reference-free detection method

The unpublished RUFUS reference-free de novo variant calling algorithm^23^ was used to call de novo variants in the probands within each of the 14 families. Rufus works by directly comparing the k-mer sequences from the raw Illumina reads between a child and his/her parents, to identify unique DNA sequence present in the child but not in the parents, thus representing de novo mutations. Sequencing reads containing such unique K-mer sequences are assembled using an in-built sequence assembler. Assembled contigs, containing the de novo mutant allele, are mapped back to the human reference sequence for localization, using the BWA-MEM^19^ algorithm. The BWA-MEM alignment files are parsed and converted to a VCF^53^ format variant report output. All types of de novo mutations (SNPs, short INDELs, and SVs of all types) are identified in a single run of the program.

#### Variant prioritization using the gene.iobio tool

Gene.iobio (http://gene.iobio.io) version 2.0 was used to identify the most likely disease-causing de novo variant in each subject. We first generated an exhaustive list of candidate genes that have been either known to harbor EIEE-causing mutations, or may plausibly harbor such mutations (see Supp. Table 4). We retrieved the list of genes part of EIEE diagnostic panel tests from four clinical diagnostic laboratories (Ambry: 100 genes, GeneDX: 87 genes, Invitae: 63 genes, and University of Chicago: 59 genes). We also used the Phenolyzer tool to generate a gene list using the phenotype term “EIEE”, and considered the top 100 genes on this list. We then merged the five lists; this resulted in our EIEE candidate gene list of 223 unique entries. Second, for each subject, we selected the RUFUS-called de novo mutation candidates using the “Files” tab from within gene.iobio. Here we also selected the sequence alignment (BAM) files for the subject (child) and both parents, so we can examine the sequence coverage at candidate de novo mutation sites. We then uploaded the list of 223 EIEE candidate genes using the “Genes” tab. Third, we activated the “Analyze all genes” button to annotate, assess the predicted impact, and rank each candidate de novo mutation in the subject. This analysis lasted a few minutes. After de novo mutations in every candidate gene are ranked, gene.iobio automatically re-sorts the genes, according to the most harmful variant present in the gene (see Fig. 3 for an illustration).

#### Mutation confirmation

All mutations we confirmed with Sanger sequencing, with the exception of the mutation predicted in *EEF1A2*. We were unable to obtain an amplicon despite multiple different PCR primer designs and PCR attempts.

### Subject diagnostic cost analysis

For determination of charges, all charges related to testing for the purpose of diagnosis were collected, including both in-patient and out-patient testing, brain MRIs, and EEGs. We determined, both manually and by computer search, all lab and radiology testing related to diagnosis. Tests included general and disease screening labs, as well as disease-specific testing (for example, respectively: blood chemistry, hemoglobin; chromosome karyotype; gene testing). Tests related to clinical patient care (such as monitoring of drug levels) and professional fees were not included. Charges for each test were only included for the first instance of that test being obtained. However, we did include charges for repeat MRIs and repeat EEGs. PCH is part of Intermountain Healthcare, and complete charge data was extracted for each subject from the electronic data warehouse and were standardized to 2013 constant US dollars.^54^

### Data availability

Sequencing data for all de-identified patients and family members will be made available through dbGaP in conjunction with publication.

### Code availability

All software used in this study is freely available and open source. We provide links to each software package below:

BWA-MEM: https://github.com/lh3/bwa

GATK: https://software.broadinstitute.org/gatk/

LUMPY: https://github.com/arq5x/lumpy-sv

RUFUS: https://github.com/jandrewrfarrell/RUFUS

GEMINI: https://github.com/arq5x/gemini

GENE.IOBIO: https://gene.iobio.io/

## Electronic supplementary material

Supplemental table

Supplemental figure 1

## Electronic supplementary material

**Supplementary information** accompanies the paper on the *npj Genomic Medicine* website (10.1038/s41525-018-0061-8).
