# Supplementary material for: Whole-genome analysis for effective clinical diagnosis and gene discovery in early infantile epileptic encephalopathy
Source: NPJ Genom Med. 2018 Aug 13;3:22. doi: 10.1038/s41525-018-0061-8 (PMC6089881; doi:10.1038/s41525-018-0061-8)
Supplement: Supplementary file 2 — Supplemental figure 1 [file 41525_2018_61_MOESM2_ESM.docx]

**SUPPLEMENTARY FIGURES**

**
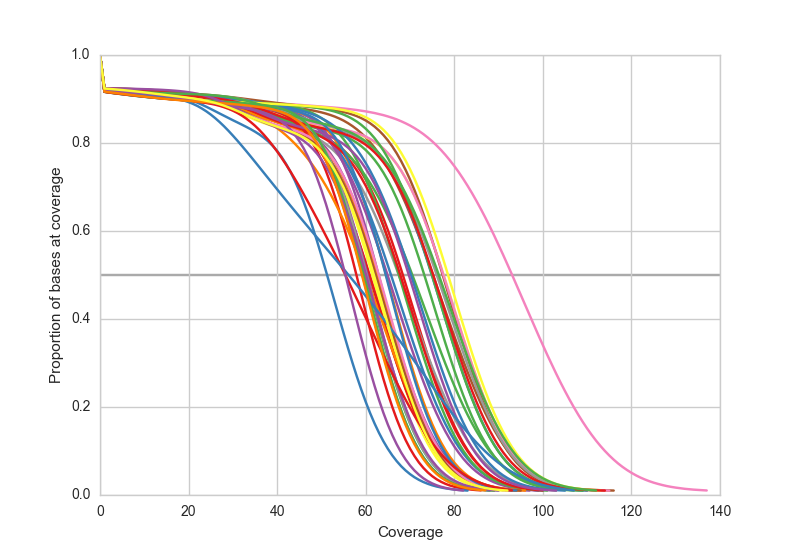
**

**Supplementary Figure 1. The cumulative distribution of genome-wide sequencing coverage for all subjects and parents.**
